# Disorder to Order Transition and Ordered Morphology of Coil-Comb Block Copolymer by Self-Consistent Field Theory

**DOI:** 10.1186/s11671-015-1035-8

**Published:** 2015-08-18

**Authors:** Zhibin Jiang, Zhiyuan Qian, Hong Yang, Rong Wang

**Affiliations:** Key Laboratory of High Performance Polymer Materials and Technology of Ministry of Education, State Key Laboratory of Coordination Chemistry and Collaborative Innovation Center of Chemistry for Life Sciences, Department of Polymer Science and Engineering, School of Chemistry and Chemical Engineering, Nanjing University, Nanjing, 210093 China

**Keywords:** Coil-comb block copolymer, Disorder to order transition, Phase separation, Self-consistent field theory

## Abstract

The disorder to order transition and the ordered patterns near the disordered state of coil-comb copolymer A-*b*-(B_*m* + 1_-*g*-C_*m*_) are investigated by the self-consistent field theory. The phase diagrams of coil-comb copolymer are obtained by varying the composition of the copolymer with the side chain number *m* = 1, 2, and 3. The disorder to order transition is far more complex compared with the comb copolymer or linear block copolymer. As the side chain number *m* increases, the Flory-Huggins interaction parameter of disorder to order transition (DOT) increases and the lowest DOT occurs when the volume fractions of blocks A, B, and C are approximately equal. When one component is the minority, the disorder to order transition curve is similar with binary copolymer, but the curve shows the asymmetric property. The comb copolymer is more stable with larger side chain number *m* and shorter side chain. The ordered patterns from the disordered state are discussed. The results are helpful for designing coil-comb copolymers and obtaining the ordered morphology.

## Background

The ability of block copolymers to spontaneously form microphase-separated structures with length scales of 10–100 nm indeed makes them extensive applications in nanoscience, such as pore materials [1], drug delivery [2], and templates [3, 4]. Recently, researchers are devoted to design block copolymers with different architectures to engineer functional materials. Even the crystal structures of block copolymers [5–9] were intensively studied. For example, the Frank-Kasper *σ* phase can form in two different single-component, sphere-forming, block copolymer melts near the order-disorder transition temperature (*T*_ODT_) [6, 7]. Xie et al. [8] studied asymmetric miktoarm block copolymers which can self-assemble into fcc, bcc, A15, and the complex *σ* phase. They even designed multiblock terpolymers which can self-assemble into various binary mesocrystals with space group symmetries of a large number of binary ionic crystals, including NaCl, CsCl, ZnS, *α*-BN, AlB_2_, CaF_2_, TiO_2_, ReO_3_, Li_3_Bi, Nb_3_Sn(A15), *α*-Al_2_O_3_, etc. [9]. This study showed that block copolymer with different architectures can be used to obtain the complex nanostructures or even construct crystal phase by macromolecular metallurgy in a mesoscale.

Coil-comb copolymers, which self-assemble at a length scale of a few nanometers, can be constructed by attaching oligomers or small molecules to polymers via covalent bond and noncovalent-bonding interactions, for example, hydrogen bonding, electrostatic interactions, or metal coordination [1, 3, 4, 10–27]. They have potential applications such as in electrical, biological, and other functional materials. Feng et al. [12–14] focused on double hydrophilic coil-comb copolymer, in which the side chain grafted to the backbone through covalent bond. And they found its potential applications in templates and biological materials. Ten Brinke and co-workers [3, 4, 16–21, 26] reported a series of A-*b*-(B_*m* + 1_-*g*-C_*m*_) coil-comb copolymers, where the small molecules C are weakly connected to backbone B through hydrogen bonds, and the coil-comb copolymers can self-assemble into hierarchically ordered structures. In these structures, there were two different length scales: the larger length scale period is mainly driven by the separation between the comb part and the coil part, whereas the small length period is produced by the segregation within the comb part.

Besides, some researchers also found these hierarchically ordered structures of coil-comb copolymer based on theoretical simulation [28, 29]. For instance, by using the real-space implemented self-consistent field theory, Wang et al. [29] reported that the coil-comb block copolymers exhibit hierarchically ordered microstructures, including parallel and perpendicular lamella-within-lamella, cylinder-within-lamella, lamella-within-cylinder, and cylinder-within-cylinder.

The ordered phase can form when the repulsive interaction parameter *χN* is larger than the interaction parameter of the disorder to order transition. Therefore, it is very important to determine the disorder to order transition of block copolymers. It can help us to find ordered microstructures easily. Therefore, more and more researchers pay attention to the disorder to order phase transition of supramolecular polymers [30–32]. At the meantime, self-consistent field theory (SCFT) method has been largely used to study the phase behavior of block copolymers [33–37]. It is also used to study the disorder to order transition of block copolymers [38, 39]. Even the SCFT method has been used to successfully study the polydispersity-induced disorder to order transition of block copolymers [33, 40–42]. In this work, we consider the disorder to order transition of coil-comb copolymer A-*b*-(B_*m* + 1_-*g*-C_*m*_) with different side chain numbers by using the SCFT. Due to the complexity of this copolymer, we only consider the stability of the homogeneous phase relative to the microseparated state. The related parameters are the side chain number *m*, the volume fraction of blocks A, B, and C, and the Flory-Huggins parameter *χN*. We construct the phase diagram of disorder to order transition of coil-comb copolymer by continuously varying the composition of the block copolymer.

## Methods

**Fig. 1:**
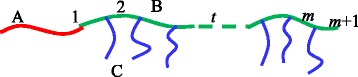
Schematic representation of a coil-comb copolymer molecule. *Red*, *green*, and *blue lines* represent main chains (*A*) and (*B*) and side block (*C*), respectively

We consider *n* comb copolymers A-b-(B_*m* + 1_-g-C_*m*_) with polymerization *N* in a volume *V* and there are *m* branching points (or the side chain number) along the main chain B, which divide the B chain as *m* + 1 equal parts (we called it “divided sections”) with polymerization *N*_B_ and each side chain C has *N*_C_ segments for block C, respectively. So, *N* = *N*_A_ + (*m* + 1)*N*_B_ *+ mN*_C_. The schematic diagram of a comb copolymer molecule is presented in Fig. 1. The monomers of the main chain and the side ones are assumed to be flexible with a statistical length *a*. Therefore, the compositions (average volume fractions) are *f*_A_ = *N*_A_/*N*, *f*_B_ = (*m* + 1)*N*_B_/*N*, *f*_C_ = 1 − *f*_A_ − *f*_B_ for blocks A, B, and C, respectively.

Due to the different architectures of blocks A, B, and C, we define six distribution functions, i.e., *q*_A_(**r**, *s*) and *q*_A_^+^(**r**, *s*), *q*_B_(**r**, *s*, *t*) and *q*_B_^+^(**r**, *s*, *t*), and *q*_C_(**r**, *s*) and *q*_C_^+^(**r**, *s*, *t*) where *s* is the contour along the main chain for AB and along the side chain for C, *t* is the number along the block B of main chain AB divided by the side chains C, and it belongs to [1, *m* + 1]. With these definitions, the polymer segment probability distributions *q* and *q*^+^ for main chain AB and side chain C satisfy the modified diffusion equations:

1 $$ \frac{\partial }{\partial s}q=\frac{N{a}^2}{6}{\nabla}^2q-wq $$

2 $$ \frac{\partial }{\partial s}{q}^{+}=-\frac{N{a}^2}{6}{\nabla}^2{q}^{+}+w{q}^{+} $$

where *w* is *w*_A_ when *s* belongs to block A, *w*_B_ when belongs to block B, and *w*_C_ when belongs to block C. The initial conditions are *q*_C_(**r**, 0) = 1, *q*_A_(**r**, 0) = 1, *q*_B_(**r**, 0, 1) = *q*_A_(**r**, *N*_A_), *q*_B_(**r**, 0, *t*) = *q*_B_(**r**, *N*_B_, *t* − 1)*q*_C_(**r**, *N*_C_) for *t* > 1, *q*_B_^+^(**r**, *N*_B_, *m* + 1) = 1, *q*_B_^+^(**r**, *N*_B_, *t*) = *q*_B_^+^(**r**, 0, *t* + 1)*q*_C_(**r**, *N*_C_) for *t* < *m* + 1, *q*_C_^+^(**r**, *N*_C_, *t*) = *q*_B_(**r**, *N*_B_, *t*)*q*_B_^+^(**r**, 0, *t* + 1), *q*_A_^+^(**r**, *N*_A_) = *q*_B_^+^(**r**, 0, 1), where *t*∈ [1, *m*]. Accordingly, the partition function of a single chain subject to the mean field *w*_*i*_, where *i* represents block species A, B, and C, can be written as *Q* = ∫*d***r***q*_B_(**r**, *N*_B_, *m* + 1).

With the above description, the free energy function (in units of *k*_*B*_*T*) of the system is given by:

3 $$ F=- \ln \left(Q/V\right)+1/V{\displaystyle \int d\mathbf{r}}\left[\frac{1}{2}{\displaystyle \sum_{i\ne j}{\chi}_{ij}N{\phi}_i{\phi}_j}-{\displaystyle \sum_i{w}_i{\phi}_i}-\xi \left(1-{\displaystyle \sum_i{\phi}_i}\right)\right] $$

where *χ*_*ij*_ is the Flory-Huggins interaction parameter between different species (*i*, *j* = A, B, C, *i* ≠ *j*), *ϕ*_*i*_ is the monomer density of each species (*i* = A, B, C), and *ξ* is the Lagrange multiplier (as a pressure).

Minimization of the free energy to mean field, density, and pressure, *δF*/*δw* = *δF*/*δϕ* = *δF*/*δξ* = 0, leads to the following self-consistent field equations that describe the equilibrium state:

4 $$ {\phi}_{\mathrm{A}}\left(\mathbf{r}\right)=\frac{V}{QN}{\displaystyle {\int}_0^{N_{\mathrm{A}}}ds{q}_{\mathrm{A}}\left(\mathbf{r},s\right){q_{\mathrm{A}}}^{+}\left(\mathbf{r},s\right)} $$

5 $$ {\phi}_{\mathrm{B}}\left(\mathbf{r}\right)=\frac{V}{QN}{\displaystyle \sum_{t=1}^{m+1}{\displaystyle {\int}_0^{N_{\mathrm{B}}}ds{q}_{\mathrm{B}}\left(\mathbf{r},s,t\right){q_{\mathrm{B}}}^{+}\left(\mathbf{r},s,t\right)}} $$

6 $$ {\phi}_{\mathrm{C}}\left(\mathbf{r}\right)=\frac{V}{QN}{\displaystyle \sum_{t=1}^m{\displaystyle {\int}_0^{N_{\mathrm{C}}}ds{q}_{\mathrm{C}}\left(\mathbf{r},s\right){q_{\mathrm{C}}}^{+}\left(\mathbf{r},s,t\right)}} $$

7 $$ {w}_{\mathrm{A}}\left(\mathbf{r}\right)={\chi}_{\mathrm{A}\mathrm{B}}N{\phi}_{\mathrm{B}}\left(\mathbf{r}\right)+{\chi}_{\mathrm{A}\mathrm{C}}N{\phi}_{\mathrm{C}}\left(\mathbf{r}\right)+\xi \left(\mathbf{r}\right) $$

8 $$ {w}_{\mathrm{B}}\left(\mathbf{r}\right)={\chi}_{\mathrm{A}\mathrm{B}}N{\phi}_{\mathrm{A}}\left(\mathbf{r}\right)+{\chi}_{\mathrm{B}\mathrm{C}}N{\phi}_{\mathrm{C}}\left(\mathbf{r}\right)+\xi \left(\mathbf{r}\right) $$

9 $$ {w}_{\mathrm{C}}\left(\mathbf{r}\right)={\chi}_{\mathrm{A}\mathrm{C}}N{\phi}_{\mathrm{A}}\left(\mathbf{r}\right)+{\chi}_{\mathrm{B}\mathrm{C}}N{\phi}_{\mathrm{B}}\left(\mathbf{r}\right)+\xi \left(\mathbf{r}\right) $$

10 $$ {\phi}_{\mathrm{A}}\left(\mathbf{r}\right)+{\phi}_{\mathrm{B}}\left(\mathbf{r}\right)+{\phi}_{\mathrm{C}}\left(\mathbf{r}\right)=1 $$

Here, we solve Eqs.(4)–(10) directly in real space by using a combinatorial screening algorithm proposed by Drolet and Fredrickson [43, 44]. Note that one must solve the diffusion equation first for *q*_C_(**r**, *s*) and *q*_A_(**r**, *s*) with initial condition *q*_C_(**r**, 0) = 1 and *q*_A_(**r**, 0) = 1, then for *q*_B_(**r**, *s*, *t*) with *q*_B_(**r**, 0, 1) = *q*_A_(**r**, *N*_A_), *q*_B_(**r**, 0, *t*) = *q*_C_(**r**, *N*_B_, *t* − 1)*q*_C_(**r**, *N*_C_) for *t* > 1 and for *q*_B_^+^(**r**, *s*, *t*) with *q*_B_^+^(**r**, *N*_B_, *m* + 1) = 1, *q*_B_^+^(**r**, *N*_B_, *t*) = *q*_B_^+^(**r**, 0, *t* + 1)*q*_C_(**r**, *N*_C_) for *t* < *m* + 1, and last for *q*_C_^+^(**r**, *s*, *t*) and *q*_A_^+^(**r**, *s*) with *q*_C_^+^(**r**, *N*_C_, *t*) = *q*_B_(**r**, *N*_B_, *t*)*q*_B_^+^(**r**, 0, *t* + 1) and *q*_A_^+^(**r**, *N*_A_) = *q*_B_^+^(**r**, 0, 1). Each iteration continues until the phases are stable. Several times are repeated by using different initial conditions to avoid the trapping in a metastable state. In addition, we also minimize the free energy with respect to the system size because it has been pointed out that the box size can influence the morphology [45]. The implementation of the self-consistent field theory is carried out in a two-dimensional *L*_*x*_ × *L*_*y*_ lattice with periodic boundary conditions.

We consider the disorder to order transition with different side chain numbers *m* and the ordered pattern near the disorder to order transition. In our calculation, we consider the condition: symmetrical interaction parameters *χ*_AB_*N = χ*_AC_*N = χ*_BC_*N = χN*. Thus, we calculate the crossover curve of the disordered phase and an (unspecified) ordered phase in terms of the normalized Flory-Huggins parameter *χN*, the relative composition, and the number of teeth in the comb *m*. By systematically changing the volume fractions of A, B, and C blocks, we can construct the component triangle phase diagrams in the entire range of the copolymer composition.

## Results and Discussion

- Phase diagrams of disorder to order transition for A-*b*-(B_*m* + 1_-*g*-C_*m*_) (*m* = 1–3)

**Fig. 2:**
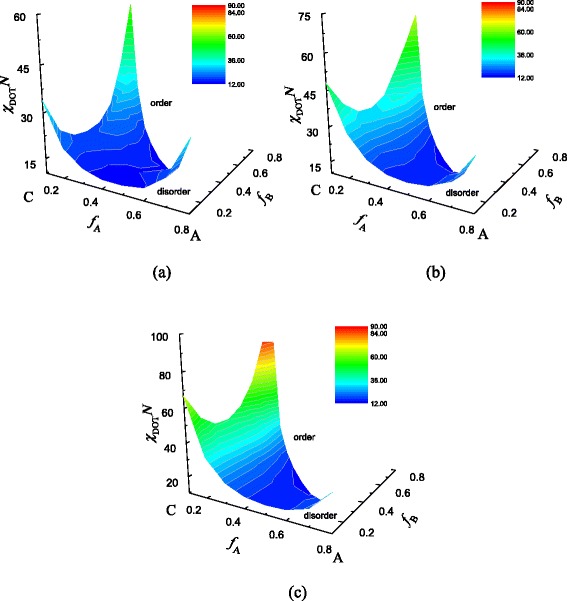
Interaction parameters of disorder to order transition *χ*_DOT_ *N* of coil-comb copolymer A-*b*-(B_*m* + 1_-*g*-C_*m*_) with different side chain numbers *m*. **a** *m* = 1. **b** *m* = 2. **c** *m* = 3. The *χ*_DOT_ *N* is represented by *colored bar scale* shown in the figure

In this part, we only consider the cases for comb side chain number *m* = 1, 2, and 3, and the interaction parameters between each block are set to be equal: *χ*_AB_*N = χ*_AC_*N = χ*_BC_*N = χN*. Besides, the chain length is *N* = 120 for these three cases. By systematically varying the volume fractions of the three blocks (*f*_A_, *f*_B_, and *f*_C_) and the Flory-Huggins parameter *χN*, we got the phase diagrams of the disorder to order transition for the coil-comb copolymer, which are shown in Fig. 2, and parts a, b, and c are referred to the situations of *m* = 1, 2, and 3, respectively. According to the figure, we can find that the disorder to order transition (DOT) parameter *χN* is larger at the corners of the three blocks, and the *χN* of DOT is lower when the volume fractions of the three blocks are approximately comparable. By comparing these three figures, we also can see that with the increasing of comb side chain number *m*, the *χN* of DOT increases largely, which corresponds to the lower temperature to microphase separate, i.e., the system becomes more stable. We can see that the *χN* of DOT is larger when the coil-comb copolymer has the longer block B or longer block C. This result is different from our previous research on the DOT of A_*m* + 1_(BC)_*m*_ [46] because the topological structure between these two kinds of copolymer is different. In this condition, block A is much free and only one end is confined by block B, while blocks B and C are much restricted by the junctions.

**Fig. 3:**
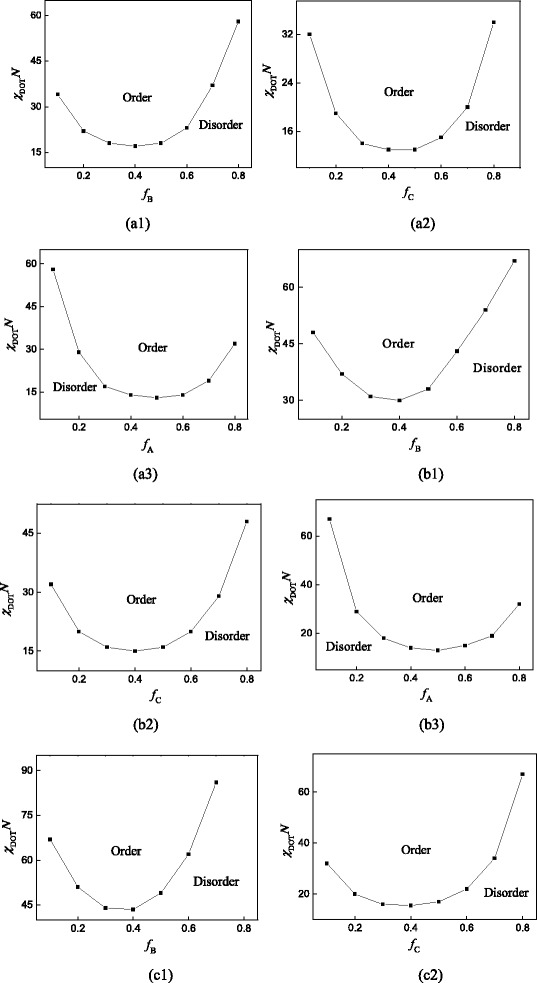
Phase diagram for coil-comb copolymer A-*b*-(B_*m* + 1_-*g*-C_*m*_) with the volume fraction of one block is small. (a1) *m* = 1, *f*_A_ = 0.1; (a2) *m* = 1, *f*_B_ = 0.1; (a3) *m* = 1, *f*_C_ = 0.1; (b1) *m* = 2, *f*_A_ = 0.1; (b2) *m* = 2, *f*_B_ = 0.1; (b3) *m* = 2, *f*_C_ = 0.1; (c1) *m* = 3, *f*_A_ = 0.1; (c2) *m* = 3, *f*_B_ = 0.1; (c3) *m* = 3, *f*_C_ = 0.1

Besides, we also got the phase diagrams when the volume fractions are *f*_A_, *f*_B_, *f*_C_ = 0.1, which are shown in Fig. 3, respectively. We can find that when the volume fraction *f*_A_, *f*_B_, or *f*_C_ is small, the diagrams are similar with those of diblock copolymer, which also has a minimum of *χN* in the phase diagram. When *f*_A_ is small, as shown in Fig. 3 (a1), (b1), (c1), the topological structure of copolymer becomes comb-like, and the sections of blocks B and C are restricted by each other. However, block B is also restricted by block A. So, the influence of block B on DOT is larger than that of block C. Compared with the comb block copolymer (B_*m* + 1_-*g*-C_*m*_) [37], we can see that the disorder to order transition is very similar. The curves are asymmetric. When *f*_B_ is small, from Fig. 3 (a2), (b2), (c2), we can see that with the increase of the side chain number *m*, the gap between the *χN* of ends in the diagram is much larger. Because with the increase of the side chain number *m*, more segments of block C are restricted, while the length of block A is still fixed and relatively free, which means the influence of block A on the DOT is smaller than that of block C. Based on Fig. 3 (a3), (b3), (c3), we can get the similar conclusion for the situation when *f*_C_ is small. According to the above analysis, we conclude that block A can separate from the copolymer first, then block C and last block B, which are determined by the topological structure of the copolymer. From Figs. 2 and 3, we can see that the confined segments increase with the increase of the side chain number, so the *χN* increases, which means the lower temperature of microphase separation.

b. Disorder to order transition for A-*b*-(B_*m* + 1_-*g*-C_*m*_) when fixing blocks A, B, and C

**Fig. 4:**
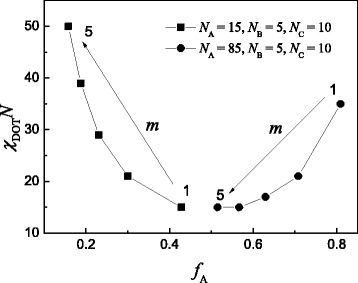
Interaction parameters of disorder to order transition *χ*_DOT_ *N* of coil-comb copolymer A-*b*-(B_*m* + 1_-*g*-C_*m*_) with different side chain numbers *m* when fixing the length of the three blocks for two cases: (1) *N*_A_ = 15, *N*_B_ = 5, *N*_C_ = 10(squares) and (2) *N*_A_ = 85, *N*_B_ = 5, *N*_C_ = 10(spheres). The *x* coordinate is represented by the volume fraction of block A converted by *f*_A_ = *N*_A_/(*N*_A_ + (*m* + 1)*N*_B_ *+ mN*_C_)

If we fix the length of the three sections (block A, block B, and side chain C), the interaction parameters of disorder to order transition vary with the increase of the side chain number *m*. Figure 4 shows the interaction parameters of disorder to order transition *χ*_DOT_*N* of coil-comb copolymer A-*b*-(B_*m* + 1_-*g*-C_*m*_) for two cases: (1) *N*_A_ = 15, *N*_B_ = 5, *N*_C_ = 10(squares) and (2) *N*_A_ = 85, *N*_B_ = 5, *N*_C_ = 10(spheres), where the *x* coordinate is represented by the volume fraction of block A converted by *f*_A_ = *N*_A_/(*N*_A_ + (*m* + 1)*N*_B_ *+ mN*_C_). When the volume fraction of coil part (block A) is less than 0.5, the *χ*_DOT_*N* increases with the increase of the side chain number *m*. But when the volume fraction of coil part (block A) is more than 0.5, the *χ*_DOT_*N* decreases with the increase of the side chain number. From the figure, we can see that the *χ*_DOT_*N* is relatively low when *f*_A_ is near to 0.5, while the *χ*_DOT_*N* increases when *f*_A_ is far from 0.5 by varying the side number *m* of the comb part.

c. Ordered pattern near the disorder to order transition

### Ordered Morphology

Based on the phase diagrams of the disorder to order transition, we also predicted the ordered pattern near the disorder to order transition. Several times are repeated by using different initial conditions to avoid the trapping in a metastable state. In addition, we also minimize the free energy with respect to the system size because it has been pointed out that the box size can influence the morphology. From the ordered pattern, we can clearly see the self-assembly structure of the coil-comb block copolymer. Here, we only present the phase diagrams for symmetrical interaction parameters, i.e., *χ*_AB_*N* = *χ*_AC_*N* = *χ*_BC_*N* = *χN*. We only consider the side chain number *m* = 1–3.

**Fig. 5:**
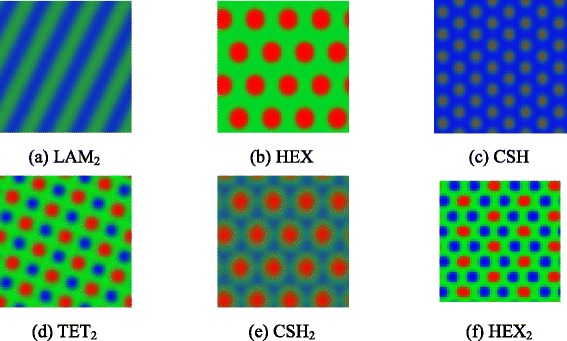
Ordered patterns of the coil-comb block copolymer. **a** LAM_2_. **b** HEX. **c** CSH. **d** TET_2_. **e** CSH_2_. **f** HEX_2_

Figure 5 shows the ordered patterns near the disorder to order transition for *m* = 1–3. The stable morphologies are two-color lamella (LAM_2_), hexagonal phase (HEX), core-shell hexagonal phase (CSH), two interpenetrating tetragonal phase (TET_2_), hexagonal lattice outside core-shell hexagonal phase (CSH_2_), and hexagonal lattice outside hexagonal phase (HEX_2_).

### Phase Diagram Based on the Ordered Pattern Near DOT

**Fig. 6:**
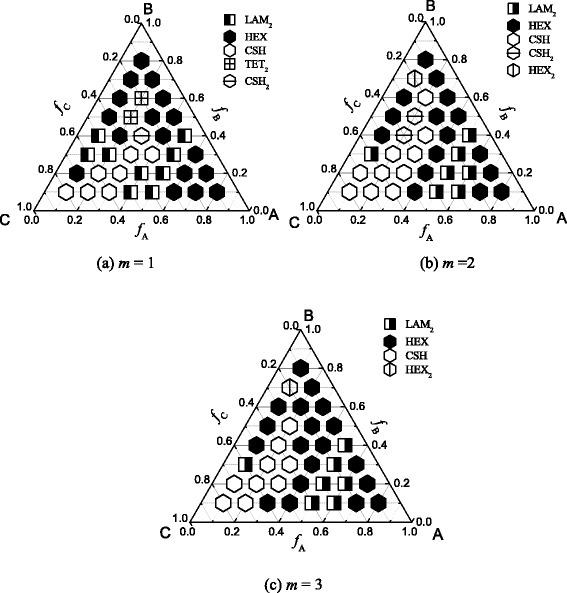
Phase diagram based on the ordered pattern near DOT. **a** *m* = 1. **b** *m* = 2. **c** *m* = 3

Comparing with the morphologies of coil-comb copolymer at larger *χN* [36], less morphologies occur near the disorder to order transition. Figure 6 presents the phase diagram based on the ordered pattern near DOT for coil-comb copolymer with teeth *m* = 1, 2, and 3. For *m* = 1, there are five ordered morphologies near the disordered state. They are LAM_2_, HEX, CSH, TET_2_, and CSH_2_. The morphologies are simple compared with those at larger interaction parameters. Near the three corners, the ordered phase is hexagonal (HEX or CSH). When one of the components is minority, the phase behavior is similar to that for diblock copolymer. But, when the three components are comparable, the stable phase is CSH, which is different from diblock copolymers. The complex phases, such as TET_2_ and CSH_2_, emerge near the disordered state. Here, we can see that near the corner C, the stable phase is CSH_2_, while the stable phase near the other two corners A and B is HEX. This result shows that longer block C easily separates from block B. The coil part (block A) can spontaneously separate from the comb part. Therefore, the core-shell hexagonal phase forms. Near the disorder to order transition, the lamellar phase is only two colored.

For *m* = 2, the phase diagram is similar with that for *m* = 1. But the region of hexagonal phase (HEX and CSH) enlarges. Another new phase (CSH_2_) occurs, and the phase TET_2_ for *m* =1 does not appear here. For *m* = 3, there are four stable phases. The complex phase CSH_2_ does not occur here, and the phase region of CSH phase enlarges. Comparing the three phase diagrams, we can see that the effect of the comb part becomes strong with the increase of the side chain number of the comb part. When the coil part block A is the minority, the phase separation of the coil-comb block copolymer is complex. The separation between the coil and comb part is natural. The separation between the main chain and the side chain will compete with the separation between the coil and comb part; therefore, the complex phases (TET_2_, CSH_2_, HEX_2_) form.

## Conclusions

The disorder to order transition and the ordered patterns from the disordered state of coil-comb copolymer A-*b*-(B_*m* + 1_-*g*-C_*m*_) are investigated by the self-consistent field theory. The phase diagrams of coil-comb copolymer are obtained by varying the composition of the copolymer with the side chain number *m* = 1, 2, and 3. The disorder to order transition is far more complex compared with the comb copolymer or linear block copolymer. As the side chain number *m* increases, the Flory-Huggins interaction parameter of disorder to order transition (DOT) increases and the lowest DOT occurs when the volume fractions of blocks A, B, and C are approximately equal. When one component is the minority, the disorder to order transition curve is similar with binary copolymer, but the curve shows the asymmetric property. The comb copolymer is more stable with larger side chain number *m* and shorter side chain. There are six ordered phases near the DOT: two-color lamellar phase (LAM_2_), hexagonal phase (HEX), core-shell hexagonal phase (CSH), two interpenetrating tetragonal phase (TET_2_), hexagonal lattice outside core-shell hexagonal phase (CSH_2_), and hexagonal lattice outside hexagonal phase (HEX_2_). The results are helpful for designing coil-comb copolymers and obtaining the ordered morphology.
